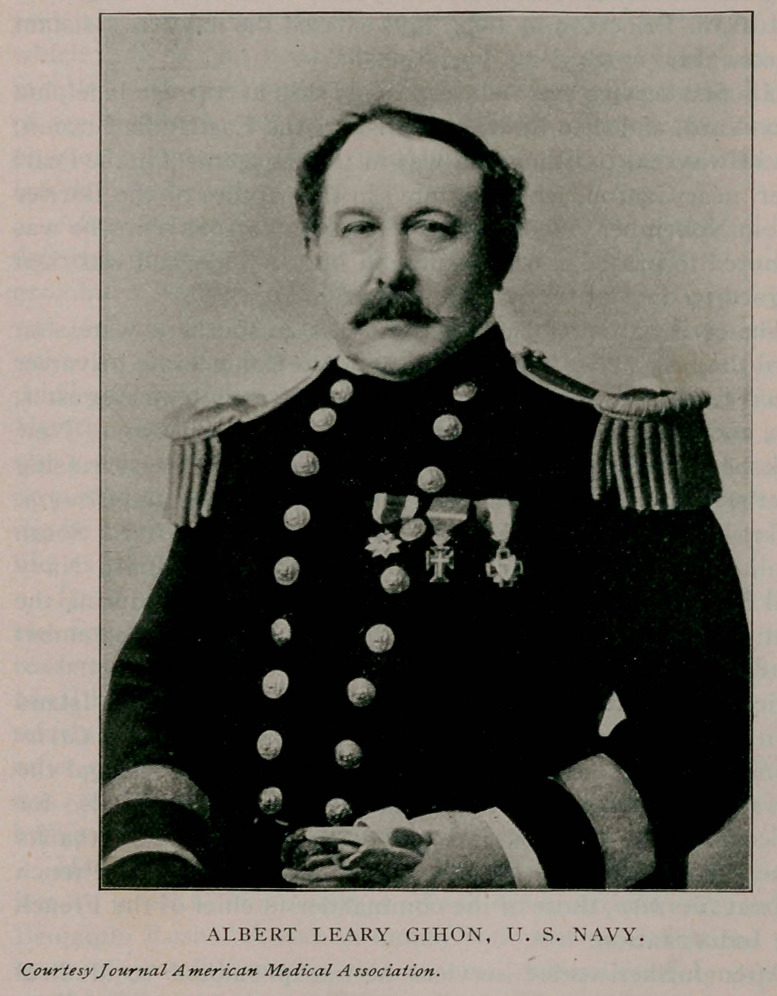# Death of Dr. Albert L. Gihon, U. S. Navy

**Published:** 1901-12

**Authors:** 


### Death of Dr. Albert L. Gihon, U. S. Navy.

DR. ALBERT LEARY GIHON, Medical Director, (Commodore) retired, died at Roosevelt Hospital of cerebral apoplexy, Sunday morning, November 17, 1902, at the age of 68 years. He never

ALBERT LEARY GIHON, U. S. NAVY.

*Courtesy Journal American Medical Association.*

recovered consciousness after the initial seizure which occurred on the morning of the previous Thursday, at his rooms at the Hotel Bayard, New York City. He was discovered prone upon the floor, insensible, about 10 a.m., and had made himself nearly ready for breakfast, as it appeared, when the attack overwhelmed him. He was removed soon afterward to the hospital by advice of Dr. Henneberger of the navy and others, who rendered every attention possible until the end came.

Dr. Gihon was born at Philadelphia, September 28, 1833; received his preliminary education at Central High School (A.B.) in that city; doctorate degree at Philadelphia College of Medicine and Surgery, 1852; was professor of chemistry and toxicolgy in his Alma Mater for two years; received the degree of A.M. from Princeton in 1854; and entered the navy as assistant surgeon May 1, 1855.

His first service was on a receiving ship at the Philadelphia Navy Yard, and then he was ordered to the East India Station; next he was sent to China and was in the engagement in the Pearl River, near Canton, which resulted in the capture of the Barrier forts in November, 1856. After service on various ships he was promoted to passed assistant surgeon May 1, 1860, and was then assigned to duty at the Brooklyn Navy Yard.

The civil war soon came and he was in southern waters on board the brig *Perry* when she captured the Confederate privateer *Savanah*, May 1, 1861. He was promoted to surgeon August 1, 1861, and after a short service at the naval rendezvous, New York, he was sent on special service in European waters, cruising after the the Confederate privateers *Alabama*, *Florida* and *Georgia*. He served late in 1864 on the blockade of the coast of South Carolina, and was senior medical officer at Portsmouth Navy Yard from 1865 to 1868. He was aboard the *Idaho* during the great typhoon, which wrecked the ship at Nagasaki, September 21, 1869.

For services rendered the Portuguese colony at Dilly, Island of Timor, and the Portuguese men-of-war, *Principe Dom Carlos* and *Sa da Bandiera*, he received from the King of Portugal the decoration of Knight of the Military Order of Christ; also for services to H. B. M. ships *Flint* and *Dawn* he received the thanks of the British Government, and for similar services to the French gunboat *Scorpion*, those of the commander-in-chief of the French East India Station.

After further varied services he was promoted to Medical Inspector, November 7, 1872, and given special duty in the Bureau of Medicine and Surgery, and soon after served on the flagships *Wabash* and *Franklin* as surgeon-of-the-fleet on the European station. He was head of the medical department at the Naval Academy at Annapolis from 1875 to 1880. He designed and superintended the construction of the hospital-ship model, exhibited at the Philadelphia Centennial Exposition, 1876, and presented at the same place the ambulance cot which bears his name and which was adopted for use in the navy. He was commissioned as medical director August 20, 1879, and subsequently served as such at the naval hospitals at Washington, Mare Island and Brooklyn, and on special duty at New York. He became senior medical director of the navy and attained the rank of Commodore May 1, 1895, after 40 years' service, during which time he had been unemployed only one year and ten months. He was retired from active service under the limitation of age, September 28, 1895, but continued to enjoy his superior physical and mental vigor until stricken with his fatal illness a few days before his death.

After his retirement he continued to represent the medical department of the Navy, as he had done since 1876, in the various prominent National Medical Societies, until recently the privilege was denied him by Surgeon-General Van Ruypen for some unknown reason. He applied to the Surgeon-General for his usual credentials to the recent meeting of the American Public Health Association at Buffalo, and was cruelly told that as he was a permanent member he could attend upon his own account, or words to that effect. He did attend and the week of the meeting he spent in Buffalo was one of the pleasantest periods of his life, as he himself expressed it.

Dr. Gihon was gifted both as a writer and speaker, and these two accomplishments were often the source of much delight to his readers and listeners. He wrote many papers and addresses on naval hygiene, public health, and on questions relating to sanitary reform, of which he had made special study, and upon many other cognate branches of medicine and surgery. In debate he was ready, courteous, able and resourceful,—sometimes even brilliantly entertaining and instructive.

He originated and carried forward to partial success the project to erect a monument at Washington to the memory of Benjamin Rush. A sum of money sufficient to ensure the ultimate success of the proposition in some form is already on deposit.

Dr. Gihon was married April 3, 1860, to Clara Montford Campfield, of Savannah, Ga. Three children, Charlotte, who died June 18, 1885, and Albert Dakin and Clarence Montford, were born of this marriage. The widow and two sons survive. The sons are both artists of prominence among the younger group of Americans at Paris. They were represented by pictures at the Pan-American Art exhibit, and at least one of the paintings has passed into the hands of a Buffalo art connoisseur.

Mrs. Gihon was in Paris on a visit to her sons during the past summer and sailed for home about the time of Dr. Gihon's apoplectic seizure, hence was in ignorance of the sad news that awaited her arrival on American shores. One of the sons, Clarence, in response to a cable followed by the next and a faster steamer, so both arrived about the same time to pay a last tribute to husband and father. It was a sad picture upon which we draw the curtain of seclusion and silence, leaving kinsfolk and near friends alone with the departed.

---

It was a source of great disappointment to Dr. Gihon's intimate friends that Mr. Cleveland did not appoint him Surgeon-General of the Navy. He was in line of promotion but a junior was given the place.

---

With all of Dr. Gihon's versatility of accomplishments, one of his chiefest attractions was his amiableness of character. He was of the sunniest of dispositions, a charming companion, a bon vivant in its best sense, a man of brilliant talents, without stain or blot or blemish of spirit, simple in manner, great in soul, and sweet in temper. Farewell, good friend. *Requiescat in pace!*

## Figures and Tables

**Figure f1:**